# Psychometric Properties of the Japanese Translation of the Detail and Flexibility Questionnaire (DFlex)

**DOI:** 10.3390/bs16060992

**Published:** 2026-06-15

**Authors:** Haruka Ito, Takeshi Atsumi, Mei Gushiken, Marion E. Roberts, Shinji Okazaki

**Affiliations:** 1 Graduate School of Comprehensive Human Sciences, University of Tsukuba, Ibaraki 305-8577, Japan; 2 Faculty of Medicine, Kyorin University, Tokyo 181-8611, Japan; 3 Faculty of Medical & Health Sciences, The University of Auckland, Auckland 1023, New Zealand; 4 Faculty of Education and Integrated Arts and Sciences, Waseda University, Tokyo 169-8050, Japan

**Keywords:** attention to detail, cognitive rigidity, measurement, autism spectrum disorder, internal structure, construct validation

## Abstract

Detailed attention and cognitive rigidity contribute to poorer social functioning and mental health. These cognitive functions can be measured using questionnaires or behavioral tasks but existing methods have limitations. The Detail and Flexibility Questionnaire (DFlex) addresses several of these limitations. This study developed a Japanese translation of the DFlex and collected valid evidence for its intended score interpretations. Sixty participants with autism spectrum disorder (ASD), 140 without ASD, and five participants who chose not to disclose whether they had an ASD diagnosis completed the Japanese version of the DFlex and the Japanese version of the Autism-Spectrum Quotient (AQ). Data from 192 participants were analyzed. Internal consistency was good as was the internal structure, except for one item. McDonald’s omega and Cronbach’s alpha demonstrated good internal consistency and item–total correlation was acceptable, except for one item. The Japanese DFlex correlated strongly with the AQ Attention to Detail and Attention Switching subscales, supporting convergent validity. Regarding known-group validity, the ASD and non-ASD groups showed significant differences on the Cognitive Rigidity and Attention to Detail subscales. Based on its reliability and internal structural validity, the Japanese DFlex provides a better understanding of ASD-related cognitive traits for both research and clinical practice.

## 1. Introduction

Some people are sensitive to minor errors in spelling or formatting, whereas others feel confused or stressed by sudden changes in their plans. These characteristics are associated with psychological constructs such as attention to detail and cognitive flexibility. They also describe the inclination to process information in detail, but often without incorporating these pieces of information into a larger contextual framework (Roberts et al., 2011). Cognitive flexibility, also referred to as set shifting, is characterized by the capacity to flexibly adjust one’s thinking style in response to changing situations or new task demands (Roberts et al., 2011). Within frameworks such as Weak Central Coherence and Enhanced Perceptual Functioning, attention to detail is described as an aspect of cognitive processing (Happé & Frith, 2006; Mottron et al., 2006). Difficulties in cognitive flexibility have mainly been studied by executive function research (Dajani & Uddin, 2015). A strong focus on details can make it more difficult to integrate information and may increase the risk of social isolation (de Jager & Condy, 2020; Stevenson et al., 2017). Reduced cognitive flexibility has been linked to behavioral challenges and poorer emotional and mental health (Hollocks et al., 2022; Lei et al., 2022). These characteristics contribute to difficulties in daily life. Therefore, assessing them is essential to understand and address such functional difficulties.

Behavioral tasks are often used to assess attention to detail and cognitive flexibility; however, they also present several challenges. For attention to detail, previous studies have shown that the Embedded Figures Test does not accurately capture global–local processing styles (De-Wit et al., 2017; Huygelier et al., 2018). In addition, the Embedded Figures Test and Navon task correlate only weakly, suggesting that attention to detail may not reflect a single unified neurocognitive trait (Chamberlain et al., 2017). Similar issues arise for cognitive flexibility. In card-sorting tasks, studies vary widely in how they conceptualize and score flexibility, resulting in inconsistent indicators across studies (Miles et al., 2021). The Trail Making Test has also been questioned, as performance appears to be strongly influenced by visual search skills rather than flexibility alone (Del Gatto et al., 2025). Such neurocognitive tasks often draw on multiple cognitive processes, making it difficult to isolate a specific construct for measurement. Self-reporting and behavioral measures that are presumed to assess the same ability often show weak convergence, which recent theoretical studies attribute to the limited reliability of many behavioral tasks and to the differences in the response processes underlying the two assessment methods (Dang et al., 2020). Psychometric instruments have a potential advantage, in that they may target intended cognitive tendencies more directly, albeit through subjective self-reporting.

The Autism-Spectrum Quotient (AQ; Baron-Cohen et al., 2001) is a frequently used instrument to evaluate attention to detail and cognitive rigidity. Individuals with autism spectrum disorder (ASD) exhibit heightened attention to detail and reduced cognitive flexibility (Gambra et al., 2024; Lage et al., 2024). The AQ includes the subscales Attention to Detail and Attention Switching, and a Japanese version of it is available (Wakabayashi et al., 2004). However, the AQ was designed as a broad screening tool for autistic traits, rather than a measure focused on these cognitive characteristics. Concerns have also been raised about the scale’s content; some items appear biased toward traits more commonly associated with the male brain (Roberts et al., 2011), and half of the 10 items assessing attention to detail focus narrowly on interest in numbers. These issues raise questions about whether the AQ adequately captures the intended constructs. Together, these findings highlight the need for a more suitable instrument for assessing attention to detail and cognitive flexibility.

An alternative questionnaire that assesses the attention to detail and cognitive flexibility is the Detail and Flexibility Questionnaire (DFlex; Roberts et al., 2011). The DFlex consists of 24 items rated on a six-point scale and was developed specifically to measure detail-focused thinking and cognitive flexibility. The original version of the DFlex scale was refined from a 54-item pilot instrument to 24 items using exploratory factor analysis and item response analysis. Support for the internal structure of the DFlex was demonstrated through a two-factor model with acceptable internal reliability and evidence of discriminant validity between the clinical and control groups (Roberts et al., 2011). In addition to the original English version (Roberts et al., 2011), an Italian version has been developed (Marchiol et al., 2020) and other translations are currently underway. However, no Japanese translation currently exists, and it remains unclear whether the same factor structure and psychometric properties can be replicated in Japan.

To address these gaps, this study developed a Japanese version of the DFlex and examined its psychometric characteristics, thereby establishing a foundation for assessing detail-oriented thinking and cognitive flexibility within a unified framework in the Japanese population.

By validating the Japanese version of the DFlex, this study supports both empirical understanding and theoretical development regarding cognitive styles. This measure also broadens opportunities to examine individual differences in cognition and informs future research and practice aimed at improving inclusive environments.

## 2. Materials and Methods

### 2.1. Participants and Sampling Procedure

We aimed to collect responses from at least 30 adults with ASD and 120 adults without an ASD diagnosis. An a priori sample size estimation for the confirmatory factor analysis (CFA) was conducted using the “A-priori Sample Size Calculator for Structural Equation Models” available on Daniel Soper’s StatCalc (n.d.) website. Based on the factor loadings reported by Marchiol et al. (2020), we assumed a medium effect size (f^2^ = 0.25). With an alpha level of 0.05, a desired statistical power of 0.80, two latent variables, and 24 observed variables specified, the required minimum sample size was estimated to be 136 participants. An a priori power analysis was conducted using G*Power 3.1 for independent-samples *t*-tests. Based on the findings of Marchiol et al. (2020) and Roberts et al. (2011), a medium-to-large effect size (Cohen’s d = 0.70) was assumed. The analysis was conducted using a one-tailed test with an alpha level of 0.05 and a desired statistical power of 0.95. Given the expected feasibility of recruitment, an unequal allocation ratio of 4:1 (non-ASD to ASD) was used. The analysis indicated that a minimum of 28 participants in the ASD group and 112 participants in the non-ASD group (N = 140) were required to detect the expected effect.

The participants were recruited through personal networks and social media platforms. Data were collected using an online survey administered via Google Forms in January 2026. Before enrolling in the study, the participants received information about the aims of the study, the voluntary basis of participation, and their right to withdraw at any time without consequences. The completion of the survey was considered as informed consent. The presence or absence of ASD was self-reported. Specifically, the participants were asked whether they had received a formal ASD diagnosis from a qualified healthcare professional. All data were kept secure on password-protected devices, with access restricted to members of the research team. The data were handled in accordance with institutional guidelines for research involving human participants. This study was approved by the Ethics Review Committee of the University of Tsukuba for Human Sciences (Approval No. Tsukuba 2025-231 A, approved on 16 December 2025).

The sample consisted of 60 participants in the ASD group, 140 in the non-ASD group, and five participants who chose not to disclose whether they had an ASD diagnosis. The mean age of each group was as follows: ASD group (M = 32.60 years, SD = 10.27, range = 19–63 years), non-ASD group (M = 35.74 years, SD = 11.59, range = 18–66 years), and the undisclosed-diagnosis group (M = 29.40 years, SD = 8.62, range = 21–43 years). The gender distributions were: ASD group (12 men [20%], 44 women [73%], 4 identifying as other [7%]), non-ASD group (45 men [32%], 92 women [66%], 1 identifying as other [0.7%], and 2 choosing “prefer not to say” [1.4%]), and the undisclosed-diagnosis group (2 men and 3 women).

The total AQ scores for each group were as follows: the non-ASD group had a mean score of 20.74 (SD = 7.90, range = 6–40), the ASD group had a mean score of 32.68 (SD = 7.82, range = 18–47), and the undisclosed-diagnosis group had a mean score of 28.80 (SD = 8.29, range = 18–38). Although some participants in the ASD group scored below the conventional AQ cut-off score of 33, ASD status in this study was determined based on self-reported formal diagnosis by a qualified healthcare professional rather than AQ scores. This is because the AQ is considered a screening measure of autistic traits rather than a diagnostic instrument. In addition, self-awareness of social characteristics may not always accurately reflect observable autistic traits. Furthermore, the AQ may not fully capture the presentation of autistic traits in women, as some items have been suggested to reflect more male-typical characteristics (Roberts et al., 2011).

### 2.2. Instruments

#### 2.2.1. Detail and Flexibility Questionnaire (DFlex), Japanese Version

The DFlex is a 24-item questionnaire designed to assess attention to detail and cognitive flexibility, with each item rated on a six-point scale indicating the degree of agreement. We obtained permission from the original author (M. E. R.) to develop a Japanese version of the DFlex. The translation process followed the recommended guidelines for instrument adaptation. Two independent translators produced initial drafts, which were then integrated, back-translated, and reviewed by the original author (Cruchinho et al., 2024). The first (H. I.) and the third (M. G.) authors each produced an independent draft translation, which was then discussed and combined into a single version. This version was back-translated by a professional translation service (Editage; Cactus Communications, Tokyo, Japan) and the back-translated items were reviewed by the original author. Based on the feedback, the first and second authors revised several items. These revised items were then back-translated, and final approval was obtained from the original author. Appendix A provides The Japanese version of DFlex.

Odd-numbered items indicated cognitive rigidity, whereas even-numbered items reflected attention to detail. Responses were scored as follows: Strongly Disagree = 1, Disagree = 2, Slightly Disagree = 3, Slightly Agree = 4, Agree = 5, and Strongly Agree = 6. We added an additional response option—“I do not understand this question”—to the original six-point scale to clarify whether the translation of the items was appropriate. Participants who selected this option were asked to provide comments. Because the responses including this option prevented the calculation of subscale scores, data from participants who selected it once were excluded from the validity analyses.

#### 2.2.2. Autism-Spectrum Quotient (AQ) Japanese Version

The AQ Japanese version (Wakabayashi et al., 2004) is a 50-item questionnaire rated on a four-point Likert scale that assesses the degree of autistic traits. It yields subscale scores for social skills, attention switching, attention to detail, communication, and imagination, and provides established cutoff values. Each subscale consists of 10 items. Higher scores indicate stronger autistic traits. Specifically, higher scores on the Attention Switching subscale reflect lower cognitive flexibility, whereas higher scores on the Attention to Detail subscale indicate a greater tendency to focus on details. The response options consist of four choices: definitely agree, slightly agree, slightly disagree, and definitely disagree (Baron-Cohen et al., 2001). In this study, the Cronbach’s alpha coefficients were 0.68, 95% CI = [0.61, 0.75] for the Attention Switching subscale and 0.66, 95% CI = [0.58, 0.73] for the Attention to Detail subscale. Permission to use the AQ-J study was obtained from the copyright holder, Sankyobo Co., Ltd. (Kyoto, Japan).

### 2.3. Analytic Plan

First, we analyzed the items for which the participants selected “I do not understand this question.” As previously mentioned, the participants who selected this option once were excluded from the reliability and validity analyses because subscale scores could not be calculated. To examine the response tendencies for each item, we calculated the means, standard deviations, and frequencies of Likert responses. Ceiling and floor effects were evaluated based on whether the sum or difference in the mean and standard deviation exceeded the upper or lower bounds of the possible response range. Range, skewness, and kurtosis were calculated for total and subscale scores. For CFA, a two-factor model, a one-factor model, and a bifactor model were specified. The analysis was conducted using WLSMV estimation. Model fit was evaluated using the Chi-square (χ^2^) statistic, comparative fit index (CFI), Tucker–Lewis index (TLI), root-mean-square error of approximation (RMSEA), and standardized root mean square residual (SRMR). The Chi-square statistic assesses the discrepancy between the model and data, with a non-significant value considered desirable. For the remaining fit indices, we followed the criteria used by Marchiol et al. (2020): CFI and TLI values greater than 0.95 were considered indicative of an acceptable fit, while values greater than 0.97 were considered a good fit. For the RMSEA, values of 0.05 or lower indicated a close fit, while values of 0.08 or lower indicated an acceptable fit. For the SRMR, values below 0.08 were regarded as indicative of a good fit. A factor loading of 0.40 or higher was considered acceptable. To assess reliability, McDonald’s omega, Cronbach’s alpha, and item–total correlations were calculated. Reliability indices were calculated using the covariance matrix. Values of McDonald’s omega and Cronbach’s alpha of 0.80 or higher were considered good, and values of 0.70 or higher were considered acceptable. Item–total correlations of 0.30 or higher were regarded as acceptable. Convergent validity was evaluated by analyzing Spearman’s rank correlations between the DFlex Cognitive Rigidity and AQ Attention Switching subscales, as well as between the DFlex Attention to Detail and AQ Attention to Detail subscales. Correlation coefficients can be interpreted as follows: values ranging from 0.00 to 0.10 indicate a negligible correlation, 0.10 to 0.39 a weak correlation, 0.40 to 0.69 a moderate correlation, 0.70 to 0.89 a strong correlation, and 0.90 to 1.00 a very strong correlation (Schober et al., 2018). To assess known-group validity, we conducted *t*-tests to compare the DFlex scores between the ASD and non-ASD groups. Participants who responded “prefer not to say” regarding their ASD diagnosis were excluded from the group comparisons. For the *t*-tests, Welch’s correction was applied when homogeneity of variance could not be assumed based on Levene’s test. Measurement invariance across groups was evaluated using multi-group CFA. Specifically, configural invariance, metric invariance, and scalar invariance were tested sequentially. In line with Maiolatesi et al. (2022), measurement invariance was considered to be violated when the decrease in CFI was 0.010 or greater and the increase in RMSEA was 0.015 or greater. The significance level for all statistical tests was set at 5%. Statistical analyses were conducted using JASP (version 0.95.4; JASP Team, Amsterdam, The Netherlands). The R code automatically generated using JASP is provided in Appendix B. The omega hierarchical value was calculated using R (Version 4.5.2; R Foundation for Statistical Computing, Vienna, Austria).

## 3. Results

### 3.1. Item Clarity

The number of participants selecting the response option “I do not understand this question” ranged from 0 to 11 across items. For Items 22 and 23, eight participants (3.7%) and 11 participants (5%) selected this option, respectively. For all other items, fewer than 2% of the participants chose this response, indicating that the translations were generally appropriate and easy to understand. For each item, we presented convergent open-ended feedback, namely comments expressed by two or more respondents. For Item 22, participants noted that the item seemed to combine two different ideas—becoming overly focused and relying on others for help—in a single sentence. For Item 23, some participants commented that they could not understand why failing to notice risks or opportunities would necessarily lead to anxiety or a sense of vulnerability, and that they found it difficult to imagine a concrete situation that fit the item. The comments suggested that the difficulty stemmed not from mistranslation but from the conceptual complexity of the items themselves. Therefore, these items were retained. A total of 192 participants with complete data were included in the statistical analyses.

### 3.2. Descriptive Statistics

**Figure 1:**
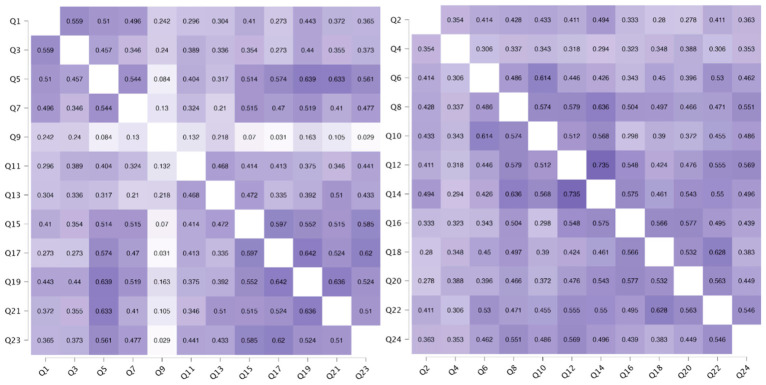
Heatmap of the inter-item correlation coefficients (**Left**: cognitive rigidity; **Right**: attention to detail).

Descriptive statistics for each item (mean, standard deviation, and frequencies of Likert responses) are presented in Table 1. No ceiling or floor effects were observed. A heatmap of the inter-item correlation coefficients is shown in Figure 1.

Total scores ranged from 38 to 144 (M = 92.20, SD = 24.42), indicating a relatively wide distribution. Cognitive rigidity scores ranged from 21 to 72 (M = 45.98, SD = 12.29), and attention to detail scores ranged from 15 to 72 (M = 46.22, SD = 13.14), both showing moderate variability. All three distributions were approximately symmetric, with skewness values close to zero (0.11, 0.21, and −0.11, respectively), and slightly platykurtic, as indicated by negative kurtosis values (−0.55, −0.59, and −0.42, respectively).

### 3.3. Internal Structure

We conducted CFA using WLSMV to examine the factor structure of DFlex. The hypothesized two-factor model showed good fit indices: χ^2^(251) = 267.51, *p* = 0.23, CFI = 0.998, TLI = 0.998, RMSEA = 0.02 (90% CI [0.00, 0.04]), and SRMR = 0.07. The standardized inter-factor correlation was very high (r = 0.95, 95% CI [0.92, 0.98]), indicating a strong association between the two latent factors. Table 2 presents the standardized factor loadings (β), along with z and p values, for the DFlex items in a two-factor model. The loading for Item 9 was not statistically significant (*p* = 0.09), whereas all other items showed significant loadings (*p* < 0.01). When the analysis was conducted with Item 9 excluded, the results were: χ^2^(229) = 233.17, *p* = 0.41, CFI = 1.00, TLI = 1.00, RMSEA = 0.01 (90% CI [0.00, 0.03]), and SRMR = 0.07.

We also conducted analyses using a one-factor model. The results were: χ^2^(252) = 273.72, *p* = 0.17, CFI = 0.998, TLI = 0.997, RMSEA = 0.02 (90% CI [0.00, 0.04]), and SRMR = 0.07. Regarding factor loadings, Item 9 showed a loading of 0.13, whereas the loadings of the other items ranged from 0.48 to 0.78. A chi-square difference test indicated that the two-factor model fit significantly better than the unidimensional model, Δχ^2^(1) = 6.21, *p* < 0.05.

We additionally tested a bifactor model, consisting of a general DFlex factor and two orthogonal specific factors corresponding to the hypothesized subdimensions. The bifactor model demonstrated acceptable model fit: χ^2^(227) = 164.30, *p* = 0.999, CFI = 1.00, TLI = 1.01, RMSEA = 0.00 (90% CI [0.00, 0.00]), and SRMR = 0.056. Regarding factor loadings, Item 17 was not significant for Factor 1; Items 12, 16, and 20 were not significant for Factor 2; and Items 3 and 9 were not significant for the general factor. The factor loadings for all other items ranged from 0.25 to 0.81.

### 3.4. Internal Consistency

For the Cognitive Rigidity subscale, McDonald’s omega was 0.89 and Cronbach’s alpha 0.89, indicating good internal consistency. Item–total correlations ranged from 0.54 to 0.73 for all items except Item 9, which showed a notably low value of 0.18. Removing Item 9 increased the alpha coefficient to 0.90, whereas removing any other item did not improve reliability. For the Attention to Detail subscale, McDonald’s omega was 0.91 and Cronbach’s alpha was 0.91, indicating strong internal consistency. Item–total correlations ranged from 0.46 to 0.75, and no item increased alpha when removed.

### 3.5. Convergent Validity

The correlations between the subscales of the DFlex and AQ are presented in Table 3. Across all participants, the Attention to Detail subscale of the DFlex showed a weak positive correlation with the AQ Attention to Detail score (r = 0.30, *p* < 0.01). Similarly, the DFlex Cognitive Rigidity subscale score was moderately and positively correlated with the AQ Attention Switching score (r = 0.67, *p* < 0.01).

### 3.6. Known-Groups Validity Evidence

To examine known-group validity, we conducted *t*-tests comparing the ASD and non-ASD groups on the DFlex Cognitive Rigidity and Attention to Detail subscales. As reference, we also conducted *t*-tests for AQ Attention to Detail and Attention Switching scores. The means, standard deviations, and test statistics for the DFlex and AQ scores are presented in Table 4. Because Levene’s test was significant for the Attention to Detail scale in the AQ, we applied Welch’s correction to all four *t*-tests. Significant group differences were found for the DFlex Cognitive Rigidity subscale (t(92.00) = 8.10, *p* < 0.01) and Attention to Detail subscale (t(115.44) = 9.81, *p* < 0.01). Regarding Cohen’s d, the effect size for the DFlex Cognitive Rigidity subscale was d = 1.33 (SE 0.20) and the Attention to Detail subscale it was d = 1.53 (SE 0.22). Significant group differences were found for the AQ Attention Switching subscale (t(115.10) = 10.01, *p* < 0.01) and Attention to Detail subscale (t(90.00) = 3.12, *p* < 0.01). Regarding Cohen’s d, the effect size for the AQ Attention Switching subscale was d = 1.56 (SE 0.22), and that for the AQ Attention to Detail subscale was d = 0.52 (SE 0.17).

Measurement invariance was examined using multi-group CFA. CFI decreased from 1.000 in the configural model to 0.969 in the metric invariance model (ΔCFI = −0.031) and further to 0.804 in the scalar invariance model (ΔCFI = −0.165). Similarly, RMSEA increased from 0.000 in the configural model to 0.058 in the metric invariance model (ΔRMSEA = +0.058) and to 0.143 in the scalar invariance model (ΔRMSEA = +0.085).

## 4. Discussion

This study aimed to evaluate the validity of the Japanese translation of the DFlex in the Japanese population and make the scale available for use in Japanese populations.

### 4.1. Construct Validity

For the two-factor model, the χ^2^ statistic was not significant, indicating no discrepancy between the model and the data. The CFI and TLI values exceeded 0.97, indicating a good model fit. The RMSEA was below 0.05, indicating a close fit, and the SRMR was below the criterion of 0.08, reflecting a good fit. A similarly good model fit has also been confirmed for the Italian version in both the eating disorder and control groups, respectively (CFI = 0.994/0.977, TLI = 0.993/0.975, RMSEA = 0.022/0.035, SRMR = 0.08/0.087; Marchiol et al., 2020). This convergence across language versions and samples supports the structural robustness of the DFlex and suggests that the factor structure is stable across cultural contexts. Regarding factor loadings, Item 9 fell below the cutoff value of 0.137; however, all other items exceeded the threshold and were considered acceptable. Neither the original English version (Roberts et al., 2011) nor the Italian version (Marchiol et al., 2020) reported Item 9 as problematic, although the factor loading for this item in the Italian version was relatively low. When we excluded Item 9 and conducted another CFA, all fit indices improved. Nevertheless, as the model fit with Item 9 was not particularly poor, it may be reasonable to retain the item to maintain consistency with the original scale and enable future cross-cultural comparisons. The content validity and cultural appropriateness of Item 9 are discussed below.

We also conducted CFA of the one-factor model. Similar to the two-factor model, the model showed a good fit, except for the low factor loading of Item 9. Previous studies reported strong associations between attention to detail and cognitive flexibility (Danner et al., 2012), suggesting that these two constructs may have also been difficult to separate clearly in the present sample. Another possible reason is the nature of self-reporting questionnaires. The DFlex does not include reverse-scored items. Individual differences in response styles—such as tendencies to give generally higher or lower ratings—may artificially inflate convergent validity and known-groups validity (İlhan et al., 2024).

The Chi-square difference test indicated that the two-factor model fit significantly better than the unidimensional model. However, the standardized inter-factor correlation was very high, suggesting a substantial overlap between the two factors.

Attention to detail is commonly regarded as a processing bias, whereas cognitive flexibility is typically conceptualized within the domain of executive functioning. Although these constructs arise from distinct theoretical frameworks, both involve attentional processes and may therefore share a common underlying factor. The bifactor model demonstrated generally favorable fit indices. Although the perfect fit indices (CFI = 1.00, TLI = 1.00) may suggest potential overfitting, the chi-square statistic was lower than the model degrees of freedom (χ^2^ = 164.3, df = 227), which mathematically results in RMSEA values of zero and TLI values greater than one. In addition, SRMR was 0.056, indicating that some residual discrepancies remained and suggesting that the model was not saturated. The omega hierarchical value (ωH = 0.91) suggested a strong general factor. Nevertheless, concerns regarding overfitting cannot be completely ruled out. In the original English version, it is recommended that the subscales be interpreted independently, and caution is advised in interpreting the total score (Roberts et al., 2011). Therefore, the validity and superiority of the bifactor model should be interpreted with caution and warrant further examination in future studies.

### 4.2. Internal Consistency

McDonald’s omega and Cronbach’s alpha values were satisfactory across both groups. However, the item–total correlation results indicated that Item 9 of the Cognitive Rigidity subscale was problematic. Item 9 reads: “I like to make plans about complex arrangements, e.g., journeys and work projects.” While adhering to a previously made plan may reflect cognitive rigidity, the mere tendency to create plans may not be a clear indicator of rigidity. To the best of our knowledge, the adaptation of this material to Japanese has not introduced culturally specific distortions or the loss of intended meaning. For all the other items, the item–total correlation indices were within acceptable ranges, suggesting that internal consistency was maintained for the rest of the scale.

### 4.3. Convergent Validity

Across the full sample, the DFlex Attention to Detail subscale correlated with the AQ Attention to Detail score, and the DFlex Cognitive Rigidity subscale correlated with the AQ Attention Switching score, indicating generally good convergent validity. However, the correlation between the DFlex Attention to Detail subscale and the AQ Attention to Detail score was weak, which is somewhat concerning, given that both scales are intended to measure the same construct. In the Italian version of the DFlex, no significant correlation was found between the Attention to Detail subscale and AQ Attention to Detail score (Marchiol et al., 2020), whereas the original English version reported a weak but significant correlation (r = 0.26, *p* < 0.01; Roberts et al., 2011). These findings suggest that the weak correlation between the Attention to Detail subscale is not specific to the Japanese translation but may instead reflect a characteristic of the scale itself. That said, considering the issues with the AQ noted in the Introduction—namely, the content inadequacy arising from the fact that half of the items in the Attention to Detail subscale assess a preoccupation with numbers—the fact that the AQ and DFlex appear to measure different aspects may be consistent with our original aims.

### 4.4. Scale Scores and Known-Groups Validity Evidence

For the control groups of previous studies, the mean DFlex scores were 34.08 (SD = 9.31) for Cognitive Rigidity and 32.80 (SD = 7.96) for Attention to Detail in the English version (Roberts et al., 2011), and 40.15 (SD = 8.98) and 33.48 (SD = 8.21), respectively, in the Italian version (Marchiol et al., 2020). By contrast, the scores in the Japanese version were 41.61 (SD = 10.00) for Cognitive Rigidity and 41.11 (SD = 11.23) for Attention to Detail. Notably, the Attention to Detail score was considerably higher than that reported for the English and Italian versions.

Based on previous findings that individuals with ASD tend to show stronger attention to detail and reduced cognitive flexibility (Gambra et al., 2024; Lage et al., 2024), we compared the DFlex scores between the ASD and non-ASD groups. Significant group differences emerged for the Attention to Detail and Cognitive Rigidity subscales, indicating that DFlex was able to distinguish between the two groups, as expected. While the original version of the questionnaire demonstrated differences between individuals with eating disorders and the control participants (Roberts et al., 2011), the present study extended these findings by showing that similar discriminative patterns were also observed between the ASD and non-ASD groups.

Regarding the AQ, the cutoff for the Attention Switching subscale is 7 points, whereas no cutoff has been established for Attention to Detail (Wakabayashi et al., 2004). In this study, the mean Attention Switching score for the ASD group was 7.55, which exceeded the cutoff, as expected, whereas the non-ASD group scored below the cutoff (M = 4.56). For Attention to Detail, the ASD group again showed higher scores (M = 5.09) compared to the non-ASD group (M = 4.14), aligning with expectations.

### 4.5. Limitations

Despite its contributions, this study has some limitations. First, it did not examine the associations with objective clinician-assessed measures. It would be informative to investigate how the Japanese DFlex relates to performance-based indices such as the Embedded Figures Test, the Navon task, card-sorting tasks, or the Trail Making Test in future research.

Second, there were no items in this questionnaire that appeared to strongly reflect cultural differences, and we included the option “I do not understand this question” to assess item comprehension among the general population. These results suggest no major issues. Nevertheless, conducting additional evaluations, such as content validity assessments by an expert panel, may help further improve the validity of the questionnaire. This may help clarify the reasons for the poor fit of Item 9.

Third, the sample size in the present study was relatively small, which may limit the generalizability of the findings. Participants were recruited online and completed the survey on a voluntary basis; therefore, the sample may be subject to self-selection bias and other forms of systematic bias. Future research should replicate these findings using larger and more diverse samples recruited through multiple methods to enhance reproducibility and generalizability.

Fourth, ASD status was determined solely on the basis of self-reported diagnoses. No independent verification was conducted using standardized diagnostic instruments such as the Autism Diagnostic Observation Schedule (ADOS) or the Autism Diagnostic Interview–Revised (ADI-R). As a result, the accuracy of the diagnostic information may be limited. In addition, the substantial heterogeneity inherent in ASD, including differences in symptom severity, was not considered, which should be acknowledged as a limitation.

Fifth, this study did not collect data on participants’ educational backgrounds; therefore, the potential influence of educational attainment on the results could not be examined. In addition, information regarding the timing of ASD diagnosis was not available. Consequently, the effects of diagnostic timing, years since diagnosis, or developmental stage at diagnosis were not taken into account.

Finally, measurement invariance was examined using multi-group CFA, and deteriorations in model fit were observed when constraints were imposed across groups from the configural level to the metric and scalar levels. According to the criteria proposed by Maiolatesi et al. (2022), these substantial changes in CFI and RMSEA indicate a lack of measurement invariance at both the metric and scalar levels. Therefore, the results of the *t*-tests comparing mean scale scores between groups should be interpreted with caution, as they may reflect comparisons between groups for which the measurement model does not fit equivalently.

## 5. Conclusions

This study evaluated the validity of the Japanese version of DFlex and demonstrated that it is a reliable and useful instrument. It thus provides an efficient means of evaluating attention to detail and cognitive flexibility, without relying on a specific modality. We hope this tool will support and expand future research on these cognitive characteristics.

## Figures and Tables

**Table 1:** Descriptive statistics.

	Mean	SD	Strongly Disagree	Disagree	Slightly Disagree	Slightly Agree	Agree	Strongly Agree
Q1	3.65	1.39	18	15	52	63	19	25
Q2	3.79	1.42	15	17	50	47	38	25
Q3	3.60	1.43	14	31	48	48	27	24
Q4	4.29	1.51	10	16	33	38	39	56
Q5	3.61	1.58	22	25	52	31	31	31
Q6	3.33	1.63	29	33	55	26	18	31
Q7	4.09	1.52	11	22	36	39	38	46
Q8	3.81	1.63	22	23	38	32	41	36
Q9	3.66	1.57	19	28	47	38	25	35
Q10	3.62	1.52	19	27	47	44	25	30
Q11	3.46	1.54	25	32	38	45	30	22
Q12	4.15	1.47	10	16	40	41	39	46
Q13	4.21	1.46	10	16	34	42	44	46
Q14	3.91	1.51	13	24	41	39	39	36
Q15	3.68	1.54	20	21	49	46	22	34
Q16	4.18	1.61	14	18	36	34	32	58
Q17	4.41	1.48	7	21	20	44	38	62
Q18	3.57	1.51	19	26	56	38	24	29
Q19	3.99	1.58	11	31	32	40	31	47
Q20	4.37	1.50	11	14	29	34	48	56
Q21	3.99	1.56	16	21	30	52	28	45
Q22	3.64	1.57	19	32	40	41	28	32
Q23	3.63	1.69	25	32	37	33	26	39
Q24	3.57	1.66	27	31	33	39	30	32

**Table 2:** Standardized factor loadings for the DFlex items in a two-factor model.

Item	Cognitive Rigidity Subscale			Attention to Detail Subscale			
	β	z	*p*	β		z	*p*
Q1	0.54	9.54	<0.001				
Q3	0.49	8.19	<0.001				
Q5	0.75	19.72	<0.001				
Q7	0.67	15.23	<0.001				
Q 9	0.14	1.69	0.091				
Q11	0.61	10.98	<0.001				
Q13	0.53	9.67	<0.001				
Q15	0.77	21.49	<0.001				
Q17	0.76	21.16	<0.001				
Q19	0.77	23.37	<0.001				
Q21	0.69	14.61	<0.001				
Q23	0.79	27.76	<0.001				
Q2				0.545	10.05		<0.001
Q4				0.508	8.86		<0.001
Q6				0.674	16.04		<0.001
Q8				0.712	17.24		<0.001
Q10				0.675	15.16		<0.001
Q12				0.719	19.36		<0.001
Q14				0.741	20.43		<0.001
Q16				0.653	13.24		<0.001
Q18				0.721	15.81		<0.001
Q20				0.716	19.90		<0.001
Q22				0.772	22.66		<0.001
Q24				0.685	15.52		<0.001

**Table 3:** Correlations among the subscales of the DFlex and AQ.

		DFlex		AQ	
		Cognitive Rigidity	Attention to Detail	Attention Switching	Attention to Detail
DFlex	Cognitive Rigidity				
	Attention to Detail	0.84 **			
AQ	Attention Switching	0.67 **	0.68 **		
	Attention to Detail	0.27 **	0.30 **	0.31 **	

** *p* < 0.01.

**Table 4:** Means and standard deviations for the DFlex and AQ.

	ASD	Non-ASD	t (df), *p*, d
DFlex (Cognitive rigidity)	55.96 (±11.53)	41.61 (±10.00)	t(92.00) = 8.10 *p* < 0.01 d = 1.33 (SE 0.20)
DFlex (Attention to detail)	57.43 (±10.05)	41.11 (±11.23)	t(115.44) = 9.81 *p* < 0.01 d = 1.53 (SE 0.22)
AQ (Attention Switching)	7.55 (±1.96)	4.56 (±2.10)	t(115.10) = 10.01 *p* < 0.01 d = 1.56 (SE 0.22)
AQ (Attention to details)	5.09 (±2.56)	4.14 (±2.16)	t(90.00) = 3.12 *p* < 0.01 d = 0.52 (SE 0.17)

## Data Availability

The dataset supporting the findings of this study is publicly available on Zenodo at https://doi.org/10.5281/zenodo.18801147 (accessed on 27 February 2026).

## Appendix A

以下にいくつかの文があります。それぞれの文について、あなた自身の状態にどの程度あてはまるか／あてはまらないかを最もよく表す回答を選択してください。私は、
(Below are a list of statements. Please circle the response that best describes to what extent you agree or disagree with each statement.)
全くあてはまらない (Strongly Disagree)
あてはまらない (Disagree)
あまりあてはまらない (Slightly Disagree)
ややあてはまる (Slightly Agree)
あてはまる (Agree)
とてもあてはまる (Strongly Agree)

**No.**	**Japanese Translation**	**Original Version**
1	他人が自分のやり方で行動してくれないと腹を立てる	I get angry if people do not do things my way.
2	ある話題について話し過ぎて、相手を退屈させてしまうことがある	I sometimes bore others as I go on to an excess about something.
3	他人の遅刻によってその日の予定を乱されると動揺する	I get upset if other people disturb my plans for the day by being late.
4	決断を下すのが苦手だ	I have difficulty making decisions.
5	他人が新しいやり方を提案すると、動揺したり落ち着かなくなったりする	When others suggest a new way of doing things, I get upset or unsettled.
6	映画・劇・本のストーリーを覚えておくのは難しいが、個々の場面を非常に詳しく覚えていられる	I find it difficult to remember the story line in films, plays or books, but can remember specific scenes in great detail.
7	一度怒りや悲しみなど感情的な状態になると、自分を落ち着かせるのがとても難しい	Once I get into an emotional state, e.g., anger or sadness, it is very difficult to soothe myself
8	重要なことも重要でないことも、同じくらいの時間をかけてしまう	I spend as much time on more or less important tasks.
9	旅程や仕事のプロジェクトなど複雑な計画を立てるのが好きだ	I like to make plans about complex arrangements, e.g., journeys and work projects.
10	文章を読むとき、全体の意味よりも細かい部分にこだわってしまう	I can get hung up on details when reading rather than understanding the gist.
11	見た目や味、感触などがいつもとほんの少しでも違うと、それに気づいて不安や不快に感じることがある	I have high levels of anxiety/discomfort: I can see/feel/taste that things might not be quite right
12	一度に一つのことに集中しすぎて、全体の状況を見失うことがある	I tend to focus on one thing at a time and get it out of proportion to the total situation.
13	物事を特定の順序や決まった手順で行うのが好きだ	I like doing things in a particular order or routine.
14	細部に気を取られて、作業の本来の目的を忘れてしまうことがある	I can get lost in details and forget the real purpose of a task.
15	ある視点から別の視点に切り替えるのが難しく、頑固でひたむきだと言われることがある	I can be called stubborn or single minded as it is difficult to shift from one point of view to another.
16	同時に複数のこと（マルチタスク）をするのが難しい	I find it difficult to do several things at once (multitasking).
17	新しい状況では、明確さやルールがないと、戸惑いやすい	I need clarity and rules when facing a new situation. Without rules, I easily feel lost.
18	状況を異なる視点から見るのが難しい	I find it hard to see different perspectives of a situation.
19	直前に予定が変更されると非常に動揺する	I get very distressed if plans get changed at the last minute.
20	細かい情報が多すぎると圧倒されることがある	I can get overwhelmed by too many details.
21	変化が嫌いだ	I dislike change.
22	私は視野が狭くなりがちなので、物事の全体像を捉えられるよう、人に助けてもらうことが多い	I depend on others to help me get things into perspective, as I tend to have a rather blinkered view on things in my life.
23	危険やチャンスに気づけないことで、不安や無防備さを感じることがよくある	I often feel vulnerable and unsafe as I am unable to see threats (or opportunities) that are out of my field of vision.
24	簡潔に書くのが苦手で、字数制限を超えてしまうことが多く、どの詳細を省くべきか判断しにくい	I find it hard to write concisely: I often overrun word limits and find it difficult to decide which details can be left out.

## Appendix B

### Appendix B.1. Descriptive Statistics

<Descriptive Statistics>
jaspDescriptives::Descriptives(
     data = NULL,
     version = “0.95”,
     formula = ~Q1 + Q2 + Q3 + Q4 + Q5 + Q6 + Q7 + Q8 + Q9 + Q10 + Q11 + Q12 + Q13 + Q14 + Q15 + Q16 + Q17 + Q18 + Q19 + Q20 + Q21 + Q22 + Q23 + Q24)
<Correlation heatmap>
jaspRegression::Correlation(
     data = NULL,
     version = “0.95”,
     heatmapPlot = TRUE,
     variables = ~Q1 + Q3 + Q5 + Q7 + Q9 + Q11 + Q13 + Q15 + Q17 + Q19 + Q21 + Q23)
jaspRegression::Correlation(
     data = NULL,
     version = “0.95”,
     heatmapPlot = TRUE,
     variables = ~Q2 + Q4 + Q6 + Q8 + Q10 + Q12 + Q14 + Q16 + Q18 + Q20 + Q22 + Q24)

### Appendix B.2. Internal Structure

<two factor model>
jaspFactor::confirmatoryFactorAnalysis(
     data = NULL,
     version = “0.95”,
     estimator = “wlsmv”,
     factors = list(list(indicators = list(types = list(“scale”, “scale”, “scale”, “scale”, “scale”, “scale”, “scale”, “scale”, “scale”, “scale”, “scale”, “scale”), value = list(“Q1”, “Q3”, “Q5”, “Q7”, “Q9”, “Q11”, “Q13”, “Q15”, “Q17”, “Q19”, “Q21”, “Q23”)), name = “Factor1”, title = “Factor 1”), list(indicators = list(types = list(“scale”, “scale”, “scale”, “scale”, “scale”, “scale”, “scale”, “scale”, “scale”, “scale”, “scale”, “scale”), value = list(“Q2”, “Q4”, “Q6”, “Q8”, “Q10”, “Q12”, “Q14”, “Q16”, “Q18”, “Q20”, “Q22”, “Q24”)), name = “Factor2”, title = “Factor 2”)),
     fitMeasures = TRUE,
     modelIdentification = “factorVariance”,
     naAction = “listwise”,
     pathPlot = TRUE,
     residualCovarianceMatrix = TRUE,
     residualsCovarying = NULL,
     standardized = “all”)
<one factor model>
jaspFactor::confirmatoryFactorAnalysis(
     data = NULL,
     version = “0.95”,
     estimator = “wlsmv”,
     factors = list(list(indicators = list(types = list(“scale”, “scale”, “scale”, “scale”, “scale”, “scale”, “scale”, “scale”, “scale”, “scale”, “scale”, “scale”, “scale”, “scale”, “scale”, “scale”, “scale”, “scale”, “scale”, “scale”, “scale”, “scale”, “scale”, “scale”), value = list(“Q1”, “Q2”, “Q3”, “Q4”, “Q5”, “Q6”, “Q7”, “Q8”, “Q9”, “Q10”, “Q11”, “Q12”, “Q13”, “Q14”, “Q15”, “Q16”, “Q17”, “Q18”, “Q19”, “Q20”, “Q21”, “Q22”, “Q23”, “Q24”)), name = “Factor1”, title = “Factor 1”)),
     fitMeasures = TRUE,
     residualsCovarying = NULL,
     standardized = “all”)
<bifactor model>
jaspSem::SEM(
     data = NULL,
     version = “0.95”,
     additionalFitMeasures = TRUE,
     estimator = “wlsmv”,
     factorScaling = “factorVariance”,
     freeParameters = NULL,
     modelTest = “standard”,
     models = list(list(name = “model 1”, syntax = list(columns = list(“JaspColumn_9_Encoded”, “JaspColumn_12_Encoded”, “JaspColumn_15_Encoded”, “JaspColumn_18_Encoded”, “JaspColumn_21_Encoded”, “JaspColumn_24_Encoded”, “JaspColumn_27_Encoded”, “JaspColumn_30_Encoded”, “JaspColumn_33_Encoded”, “JaspColumn_36_Encoded”, “JaspColumn_39_Encoded”, “JaspColumn_42_Encoded”, “JaspColumn_45_Encoded”, “JaspColumn_48_Encoded”, “JaspColumn_51_Encoded”, “JaspColumn_54_Encoded”, “JaspColumn_57_Encoded”, “JaspColumn_60_Encoded”, “JaspColumn_63_Encoded”, “JaspColumn_66_Encoded”, “JaspColumn_69_Encoded”, “JaspColumn_72_Encoded”, “JaspColumn_75_Encoded”, “JaspColumn_78_Encoded”), model = “Factor1 = ~JaspColumn_9_Encoded + JaspColumn_60_Encoded + JaspColumn_66_Encoded + JaspColumn_72_Encoded + JaspColumn_78_Encoded + JaspColumn_15_Encoded + JaspColumn_21_Encoded + JaspColumn_27_Encoded + JaspColumn_33_Encoded + JaspColumn_39_Encoded + JaspColumn_48_Encoded + JaspColumn_54_Encoded
Factor2 = ~JaspColumn_42_Encoded + JaspColumn_63_Encoded + JaspColumn_69_Encoded + JaspColumn_75_Encoded + JaspColumn_12_Encoded + JaspColumn_18_Encoded + JaspColumn_24_Encoded + JaspColumn_30_Encoded + JaspColumn_36_Encoded + JaspColumn_45_Encoded + JaspColumn_51_Encoded + JaspColumn_57_Encoded
* *
  General = ~JaspColumn_9_Encoded + JaspColumn_42_Encoded + JaspColumn_60_Encoded + JaspColumn_63_Encoded + JaspColumn_66_Encoded + JaspColumn_69_Encoded + JaspColumn_72_Encoded + JaspColumn_75_Encoded + JaspColumn_78_Encoded + JaspColumn_12_Encoded + JaspColumn_15_Encoded + JaspColumn_18_Encoded + JaspColumn_21_Encoded + JaspColumn_24_Encoded + JaspColumn_27_Encoded + JaspColumn_30_Encoded + JaspColumn_33_Encoded + JaspColumn_36_Encoded + JaspColumn_39_Encoded + JaspColumn_45_Encoded + JaspColumn_48_Encoded + JaspColumn_51_Encoded + JaspColumn_54_Encoded + JaspColumn_57_Encoded
* *
  General = ~1
“, modelOriginal = “Factor1 = ~Q1 + Q3 + Q5 + Q7 + Q9 + Q11 + Q13 + Q15 + Q17 + Q19 + Q21 + Q23
Factor2 = ~Q2 + Q4 + Q6 + Q8 + Q10 + Q12 + Q14 + Q16 + Q18 + Q20 + Q22 + Q24
* *
  General = ~Q1 + Q2 + Q3 + Q4 + Q5 + Q6 + Q7 + Q8 + Q9 + Q10 + Q11 + Q12 + Q13 + Q14 + Q15 + Q16 + Q17 + Q18 + Q19 + Q20 + Q21 + Q22 + Q23 + Q24
* *
  General = ~1
“, optionKey = “value”, prefixedColumns = list(data. = list()), types = list(“scale”, “scale”, “scale”, “scale”, “scale”, “scale”, “scale”, “scale”, “scale”, “scale”, “scale”, “scale”, “scale”, “scale”, “scale”, “scale”, “scale”, “scale”, “scale”, “scale”, “scale”, “scale”, “scale”, “scale”), value = list(“Q1”, “Q10”, “Q11”, “Q12”, “Q13”, “Q14”, “Q15”, “Q16”, “Q17”, “Q18”, “Q19”, “Q2”, “Q20”, “Q21”, “Q22”, “Q23”, “Q24”, “Q3”, “Q4”, “Q5”, “Q6”, “Q7”, “Q8”, “Q9”)))),
     naAction = “listwise”,
     pathPlot = TRUE,
     standardizedEstimate = TRUE)
<omega hierarchical (calculated using R)>
library(lavaan)
library(semTools)
dat <- read.csv(“DFlex_20260127_DFlex0419_2_KnownGroup.csv”)
modBf <- “Factor1 = ~Q1 + Q3 + Q5 + Q7 + Q9 + Q11 + Q13 + Q15 + Q17 + Q19 + Q21 + Q23
Factor2 = ~Q2 + Q4 + Q6 + Q8 + Q10 + Q12 + Q14 + Q16 + Q18 + Q20 + Q22 + Q24
General = ~Q1 + Q2 + Q3 + Q4 + Q5 + Q6 + Q7 + Q8 + Q9 + Q10 + Q11 + Q12 + Q13 + Q14 + Q15 + Q16 + Q17 + Q18 + Q19 + Q20 + Q21 + Q22 + Q23 + Q24
General = ~1”
fitBf <- cfa(modBf, data = dat, std.lv = T, estimator = ‘WLSMV’, orthogonal = T)
rel <- reliability(fitBf)
print(rel)

### Appendix B.3. Internal Consistency

It could not be output.

### Appendix B.4. Convergent Validity

jaspRegression::Correlation(
     data = NULL,
     version = “0.95”,
     pearson = FALSE,
     spearman = TRUE,
     variables = ~‘DFlex_Cognitive rigidity’ + ‘DFlex_Attention to detail’ + ‘AQ_Attention switching’ + ‘AQ_Attention to detail’)

### Appendix B.5. Known-Groups Validity Evidence

jaspTTests::TTestIndependentSamples(
     data = NULL,
     version = “0.95”,
     formula = ~‘DFlex_Cognitive rigidity’ + ‘DFlex_Attention to detail’ + ‘AQ_Attention switching’ + ‘AQ_Attention to detail,’
     effectSize = TRUE,
     equalityOfVariancesTest = TRUE,
     equalityOfVariancesTestType = “levene”,
     group = ~ group,
     student = FALSE,
     welch = TRUE)
<measurement invariance analyses>
jaspSem::SEM(
     data = NULL,
     version = “0.95”,
     additionalFitMeasures = TRUE,
     estimator = “wlsmv”,
     freeParameters = NULL,
     group = list(“”, “ID”, “age”, “gender”, “group”, “Q1”, “Q2”, “Q3”, “Q4”, “Q5”, “Q6”, “Q7”, “Q8”, “Q9”, “Q10”, “Q11”, “Q12”, “Q13”, “Q14”, “Q15”, “Q16”, “Q17”, “Q18”, “Q19”, “Q20”, “Q21”, “Q22”, “Q23”, “Q24”, “Cognitive.rigidity”, “Attention.to.detail”, “Total”),
     meanStructure = TRUE,
     modelTest = “standard”,
     models = list(list(name = “model 1”, syntax = list(columns = list(“JaspColumn_9_Encoded”, “JaspColumn_12_Encoded”, “JaspColumn_15_Encoded”, “JaspColumn_18_Encoded”, “JaspColumn_21_Encoded”, “JaspColumn_24_Encoded”, “JaspColumn_27_Encoded”, “JaspColumn_30_Encoded”, “JaspColumn_33_Encoded”, “JaspColumn_36_Encoded”, “JaspColumn_39_Encoded”, “JaspColumn_42_Encoded”, “JaspColumn_45_Encoded”, “JaspColumn_48_Encoded”, “JaspColumn_51_Encoded”, “JaspColumn_54_Encoded”, “JaspColumn_57_Encoded”, “JaspColumn_60_Encoded”, “JaspColumn_63_Encoded”, “JaspColumn_66_Encoded”, “JaspColumn_69_Encoded”, “JaspColumn_72_Encoded”, “JaspColumn_75_Encoded”, “JaspColumn_78_Encoded”), model = “Factor1 = ~JaspColumn_9_Encoded + JaspColumn_60_Encoded + JaspColumn_66_Encoded + JaspColumn_72_Encoded + JaspColumn_78_Encoded + JaspColumn_15_Encoded + JaspColumn_21_Encoded + JaspColumn_27_Encoded + JaspColumn_33_Encoded + JaspColumn_39_Encoded + JaspColumn_48_Encoded + JaspColumn_54_Encoded
Factor2 = ~JaspColumn_42_Encoded + JaspColumn_63_Encoded + JaspColumn_69_Encoded + JaspColumn_75_Encoded + JaspColumn_12_Encoded + JaspColumn_18_Encoded + JaspColumn_24_Encoded + JaspColumn_30_Encoded + JaspColumn_36_Encoded + JaspColumn_45_Encoded + JaspColumn_51_Encoded + JaspColumn_57_Encoded”, modelOriginal = “Factor1 = ~Q1 + Q3 + Q5 + Q7 + Q9 + Q11 + Q13 + Q15 + Q17 + Q19 + Q21 + Q23
Factor2 = ~Q2 + Q4 + Q6 + Q8 + Q10 + Q12 + Q14 + Q16 + Q18 + Q20 + Q22 + Q24”, optionKey = “value”, prefixedColumns = list(data. = list()), types = list(“scale”, “scale”, “scale”, “scale”, “scale”, “scale”, “scale”, “scale”, “scale”, “scale”, “scale”, “scale”, “scale”, “scale”, “scale”, “scale”, “scale”, “scale”, “scale”, “scale”, “scale”, “scale”, “scale”, “scale”), value = list(“Q1”, “Q10”, “Q11”, “Q12”, “Q13”, “Q14”, “Q15”, “Q16”, “Q17”, “Q18”, “Q19”, “Q2”, “Q20”, “Q21”, “Q22”, “Q23”, “Q24”, “Q3”, “Q4”, “Q5”, “Q6”, “Q7”, “Q8”, “Q9”))), list(name = “model 2”, syntax = list(columns = list(“JaspColumn_9_Encoded”, “JaspColumn_12_Encoded”, “JaspColumn_15_Encoded”, “JaspColumn_18_Encoded”, “JaspColumn_21_Encoded”, “JaspColumn_24_Encoded”, “JaspColumn_27_Encoded”, “JaspColumn_30_Encoded”, “JaspColumn_33_Encoded”, “JaspColumn_36_Encoded”, “JaspColumn_39_Encoded”, “JaspColumn_42_Encoded”, “JaspColumn_45_Encoded”, “JaspColumn_48_Encoded”, “JaspColumn_51_Encoded”, “JaspColumn_54_Encoded”, “JaspColumn_57_Encoded”, “JaspColumn_60_Encoded”, “JaspColumn_63_Encoded”, “JaspColumn_66_Encoded”, “JaspColumn_69_Encoded”, “JaspColumn_72_Encoded”, “JaspColumn_75_Encoded”, “JaspColumn_78_Encoded”), model = “Factor1 = ~c1 * JaspColumn_9_Encoded + c2 * JaspColumn_60_Encoded + c3 * JaspColumn_66_Encoded + c4 * JaspColumn_72_Encoded + c5 * JaspColumn_78_Encoded + c6 * JaspColumn_15_Encoded + c7 * JaspColumn_21_Encoded + c8 * JaspColumn_27_Encoded + c9 * JaspColumn_33_Encoded + c10 * JaspColumn_39_Encoded + c11 * JaspColumn_48_Encoded + c12 * JaspColumn_54_Encoded
Factor2 = ~a1 * JaspColumn_42_Encoded + a2 * JaspColumn_63_Encoded + a3 * JaspColumn_69_Encoded + a4 * JaspColumn_75_Encoded + a5 * JaspColumn_12_Encoded + a6 * JaspColumn_18_Encoded + a7 * JaspColumn_24_Encoded + a8 * JaspColumn_30_Encoded + a9 * JaspColumn_36_Encoded + a10 * JaspColumn_45_Encoded + a11 * JaspColumn_51_Encoded + a12 * JaspColumn_57_Encoded”, modelOriginal = “Factor1 = ~c1 * Q1 + c2 * Q3 + c3 * Q5 + c4 * Q7 + c5 * Q9 + c6 * Q11 + c7 * Q13 + c8 * Q15 + c9 * Q17 + c10 * Q19 + c11 * Q21 + c12 * Q23
Factor2 = ~a1 * Q2 + a2 * Q4 + a3 * Q6 + a4 * Q8 + a5 * Q10 + a6 * Q12 + a7 * Q14 + a8 * Q16 + a9 * Q18 + a10 * Q20 + a11 * Q22 + a12 * Q24”, optionKey = “value”, prefixedColumns = list(data. = list()), types = list(“scale”, “scale”, “scale”, “scale”, “scale”, “scale”, “scale”, “scale”, “scale”, “scale”, “scale”, “scale”, “scale”, “scale”, “scale”, “scale”, “scale”, “scale”, “scale”, “scale”, “scale”, “scale”, “scale”, “scale”), value = list(“Q1”, “Q10”, “Q11”, “Q12”, “Q13”, “Q14”, “Q15”, “Q16”, “Q17”, “Q18”, “Q19”, “Q2”, “Q20”, “Q21”, “Q22”, “Q23”, “Q24”, “Q3”, “Q4”, “Q5”, “Q6”, “Q7”, “Q8”, “Q9”))), list(name = “model 3”, syntax = list(columns = list(“JaspColumn_9_Encoded”, “JaspColumn_12_Encoded”, “JaspColumn_15_Encoded”, “JaspColumn_18_Encoded”, “JaspColumn_21_Encoded”, “JaspColumn_24_Encoded”, “JaspColumn_27_Encoded”, “JaspColumn_30_Encoded”, “JaspColumn_33_Encoded”, “JaspColumn_36_Encoded”, “JaspColumn_39_Encoded”, “JaspColumn_42_Encoded”, “JaspColumn_45_Encoded”, “JaspColumn_48_Encoded”, “JaspColumn_51_Encoded”, “JaspColumn_54_Encoded”, “JaspColumn_57_Encoded”, “JaspColumn_60_Encoded”, “JaspColumn_63_Encoded”, “JaspColumn_66_Encoded”, “JaspColumn_69_Encoded”, “JaspColumn_72_Encoded”, “JaspColumn_75_Encoded”, “JaspColumn_78_Encoded”), model = “Factor1 = ~c1 * JaspColumn_9_Encoded + c2 * JaspColumn_60_Encoded + c3 * JaspColumn_66_Encoded + c4 * JaspColumn_72_Encoded + c5 * JaspColumn_78_Encoded + c6 * JaspColumn_15_Encoded + c7 * JaspColumn_21_Encoded + c8 * JaspColumn_27_Encoded + c9 * JaspColumn_33_Encoded + c10 * JaspColumn_39_Encoded + c11 * JaspColumn_48_Encoded + c12 * JaspColumn_54_Encoded
Factor2 = ~a1 * JaspColumn_42_Encoded + a2 * JaspColumn_63_Encoded + a3 * JaspColumn_69_Encoded + a4 * JaspColumn_75_Encoded + a5 * JaspColumn_12_Encoded + a6 * JaspColumn_18_Encoded + a7 * JaspColumn_24_Encoded + a8 * JaspColumn_30_Encoded + a9 * JaspColumn_36_Encoded + a10 * JaspColumn_45_Encoded + a11 * JaspColumn_51_Encoded + a12 * JaspColumn_57_Encoded
  * *
JaspColumn_9_Encoded ~ a*1
JaspColumn_42_Encoded ~ b*1
JaspColumn_60_Encoded ~ c*1
JaspColumn_63_Encoded ~ d*1
JaspColumn_66_Encoded ~ e*1
JaspColumn_69_Encoded ~ f*1
JaspColumn_72_Encoded ~ g*1
JaspColumn_75_Encoded ~ h*1
JaspColumn_78_Encoded ~ i*1
JaspColumn_12_Encoded ~ j*1
JaspColumn_15_Encoded ~ k*1
JaspColumn_18_Encoded ~ l*1
JaspColumn_21_Encoded ~ m*1
JaspColumn_24_Encoded ~ n*1
JaspColumn_27_Encoded ~ o*1
JaspColumn_30_Encoded ~ p*1
JaspColumn_33_Encoded ~ q*1
JaspColumn_36_Encoded ~ r*1
JaspColumn_39_Encoded ~ s*1
JaspColumn_45_Encoded ~ t*1
JaspColumn_48_Encoded ~ u*1
JaspColumn_51_Encoded ~ v*1
JaspColumn_54_Encoded ~ w*1
JaspColumn_57_Encoded ~ x*1”, modelOriginal = “Factor1 = ~c1 * Q1 + c2 * Q3 + c3 * Q5 + c4 * Q7 + c5 * Q9 + c6 * Q11 + c7 * Q13 + c8 * Q15 + c9 * Q17 + c10 * Q19 + c11 * Q21 + c12 * Q23
Factor2 = ~a1 * Q2 + a2 * Q4 + a3 * Q6 + a4 * Q8 + a5 * Q10 + a6 * Q12 + a7 * Q14 + a8 * Q16 + a9 * Q18 + a10 * Q20 + a11 * Q22 + a12 * Q24
* *
  Q1 ~ a*1
Q2 ~ b*1
Q3 ~ c*1
Q4 ~ d*1
Q5 ~ e*1
Q6 ~ f*1
Q7 ~ g*1
Q8 ~ h*1
Q9 ~ i*1
Q10 ~ j*1
Q11 ~ k*1
Q12 ~ l*1
Q13 ~ m*1
Q14 ~ n*1
Q15 ~ o*1
Q16 ~ p*1
Q17 ~ q*1
Q18 ~ r*1
Q19 ~ s*1
Q20 ~ t*1
Q21 ~ u*1
Q22 ~ v*1
Q23 ~ w*1
Q24 ~ x*1”, optionKey = “value”, prefixedColumns = list(data. = list()), types = list(“scale”, “scale”, “scale”, “scale”, “scale”, “scale”, “scale”, “scale”, “scale”, “scale”, “scale”, “scale”, “scale”, “scale”, “scale”, “scale”, “scale”, “scale”, “scale”, “scale”, “scale”, “scale”, “scale”, “scale”), value = list(“Q1”, “Q10”, “Q11”, “Q12”, “Q13”, “Q14”, “Q15”, “Q16”, “Q17”, “Q18”, “Q19”, “Q2”, “Q20”, “Q21”, “Q22”, “Q23”, “Q24”, “Q3”, “Q4”, “Q5”, “Q6”, “Q7”, “Q8”, “Q9”)))),
naAction = “listwise”)
(model 1)
Factor1 = ~Q1 + Q3 + Q5 + Q7 + Q9 + Q11 + Q13 + Q15 + Q17 + Q19 + Q21 + Q23
Factor2 = ~Q2 + Q4 + Q6 + Q8 + Q10 + Q12 + Q14 + Q16 + Q18 + Q20 + Q22 + Q24
(model 2)
Factor1 = ~c1 * Q1 + c2 * Q3 + c3 * Q5 + c4 * Q7 + c5 * Q9 + c6 * Q11 + c7 * Q13 + c8 * Q15 + c9 * Q17 + c10 * Q19 + c11 * Q21 + c12 * Q23
Factor2 = ~a1 * Q2 + a2 * Q4 + a3 * Q6 + a4 * Q8 + a5 * Q10 + a6 * Q12 + a7 * Q14 + a8 * Q16 + a9 * Q18 + a10 * Q20 + a11 * Q22 + a12 * Q24
(model 3)
Factor1 = ~c1 * Q1 + c2 * Q3 + c3 * Q5 + c4 * Q7 + c5 * Q9 + c6 * Q11 + c7 * Q13 + c8 * Q15 + c9 * Q17 + c10 * Q19 + c11 * Q21 + c12 * Q23
Factor2 = ~a1 * Q2 + a2 * Q4 + a3 * Q6 + a4 * Q8 + a5 * Q10 + a6 * Q12 + a7 * Q14 + a8 * Q16 + a9 * Q18 + a10 * Q20 + a11 * Q22 + a12 * Q24
Q1 ~ a*1
Q2 ~ b*1
Q3 ~ c*1
Q4 ~ d*1
Q5 ~ e*1
Q6 ~ f*1
Q7 ~ g*1
Q8 ~ h*1
Q9 ~ i*1
Q10 ~ j*1
Q11 ~ k*1
Q12 ~ l*1
Q13 ~ m*1
Q14 ~ n*1
Q15 ~ o*1
Q16 ~ p*1
Q17 ~ q*1
Q18 ~ r*1
Q19 ~ s*1
Q20 ~ t*1
Q21 ~ u*1
Q22 ~ v*1
Q23 ~ w*1
Q24 ~ x*1
